# Bayesian Rare Variant Analysis Identifies Novel Schizophrenia Putative Risk Genes

**DOI:** 10.3390/jpm14080822

**Published:** 2024-08-02

**Authors:** Shengtong Han

**Affiliations:** School of Dentistry, Marquette University, Milwaukee, WI 53201-1881, USA; shengtong.han@marquette.edu

**Keywords:** rare variants, MIRAGE, SCHEMA, schizophrenia

## Abstract

The genetics of schizophrenia is so complex that it involves both common variants and rare variants. Rare variant association studies of schizophrenia are challenging because statistical methods for rare variant analysis are under-powered due to the rarity of rare variants. The recent Schizophrenia Exome meta-analysis (SCHEMA) consortium, the largest consortium in this field to date, has successfully identified 10 schizophrenia risk genes from ultra-rare variants by burden test, while more risk genes remain to be discovered by more powerful rare variant association test methods. In this study, we use a recently developed Bayesian rare variant association method that is powerful for detecting sparse rare risk variants that implicates 88 new candidate risk genes associated with schizophrenia from the SCHEMA case–control sample. These newly identified genes are significantly enriched in autism risk genes and GO enrichment analysis indicates that new candidate risk genes are involved in mechanosensory behavior, regulation of cell size, neuron projection morphogenesis, and plasma-membrane-bounded cell projection morphogenesis, that may provide new insights on the etiology of schizophrenia.

## 1. Introduction

Schizophrenia (SCZ for short) is an early-onset and complex highly heritable (about 80%) psychiatric disorder [1] that affects about 0.32% of the population worldwide among adults [2], with major symptoms including hallucinations, delusions, thought disorders, reduced expression of emotions, difficulty in social relationships, and motor and cognitive impairment. The etiology of schizophrenia remains largely elusive and is believed to be involved in both genetic and environment factors. Previous studies show significant genetic evidence from sequence data [3,4,5,6]. For example, a recent genome-wide association study (GWAS) has identified 287 common risk loci that led to the discovery of 120 genes that were pinpointed through fine-mapping [7]. Although the studies suggested that most of the heritability of schizophrenia could be contributed by common alleles, rare or ultra-rare single nucleotide variants (SNV) and copy number variation (CNV) also play a role in conferring the risk to this disorder [5,6,8,9]. For instance, in [9], 32 genes are implicated at an FDR (false discovery rate) of <5%, including 10 genes with exome-wide significance from ultra-rare variants (URV) by meta-analyzing the whole exome of 24,248 cases and 97,322 controls collected from different populations globally. In that study, the traditional burden test that is usually under-powered for rare variant analysis was used for risk gene detection. Due to the low number of rare variants, the burden test attempts to collapse a set of rare variants in a region as a single score; it tests the association between this single score and the phenotype to boost the the statistical power. The burden test is powerful when most of the rare variants to be aggregated are true risk variants, but significantly loses its power when there are few risk variants in the collapsing pool. However, many lines of evidence suggest that there could be a sparse number of rare variants associated with phenotypical traits, while most of them have little or no functional effect on disease risk [9,10,11], which poses a great challenge for rare risk variant identification.

To account for the uncertainty that a rare variant could be either a risk variant or non-risk variant and leverage variant functional annotations, we proposed a Bayesian framework—MIRAGE (mixture-model-based rare variant analysis on genes) [12], which is a powerful tool in detecting sparse rare risk variants for the following reasons: (1) the uncertainty of a rare variant (a gene) being a risk variant (gene) is captured by a probabilistic mixture model; (2) rare variants with similar effect sizes reflected by their functional annotations are aggregated into the same group to increase the power. Thanks to its sensitivity in detecting sparse rare risk variants, MIRAGE has been successful in detecting risk genes driven by sparse rare risk variants associated with autism [12], i.e., newly identified novel risk genes could be driven by one or two large effect rare variants that are not captured by other methods. Furthermore, MIRAGE only requires the total count of rare variants in cases and controls, different from SKAT [13], that needs individual-level genotype and phenotype information; this is sometimes hard or even impossible to access, making it more applicable in genetic association studies. STAAR [14], another recently proposed rare variant association test method, also needs variant collapsing when minor allele counts (MAC) are too sparse, e.g., MAC < 5; this is in the same spirit as the burden test, which will potentially cause a loss of power, as discussed before. In this paper, we use MIRAGE to analyze the ultra-rare variants in the SCHEMA case–control data [9], with the aim of identifying the novel risk genes associated with schizophrenia. In addition, significant burden is observed after excluding 32 FDR risk genes in the SCHEMA results, suggesting that more schizophrenia risk genes from rare variants remain to be discovered [9]; this also motivates the use of more powerful rare variant association testing method under the current limited sample size. Therefore, our hypothesis is that new SCZ-associated genes will be discovered by utilizing MIRAGE.

## 2. Materials and Methods

### 2.1. SCHEMA Sample

SCHEMA (Schizophrenia Exome meta-analysis) consortium, launched in 2017, aims to identify schizophrenia related genes by meta-analyzing exomes (protein coding region) aggregated from different studies globally; to date, it is the largest consortium of schizophrenia exome studies. The latest released sample has 24,248 schizophrenia cases and 50,437 internal controls collected from eleven global study sites [9], available at https://schema.broadinstitute.org/ (accessed on 2 May 2023). To boost the power of detecting novel schizophrenia genes, they incorporate 46,885 external control samples from gnomAD [9], resulting in a total of 24,248 cases and 97,322 controls. The external control sample has total count of rare variants only, i.e., with no access to individual subject level information. To minimize the effect of the artifacts, all samples were pre-processed and jointly called using the same pipeline to generate high-quality well-matched case–control samples, which are well suited for MIRAGE. In [9], they also incorporated a small proportion of de novo samples that will not be included in our analysis because MIRAGE does not yet have the functionality to handle de novo mutations [12].

### 2.2. Applying MIRAGE on SCHEMA Sample

The main challenge in the rare variant association test is the rare number of variants; as a result, many methods suffer the loss of power. Collapsing variants together is a natural way to boost statistical power, such as burden test. To adjust for the covariates effect, SKAT [13], its variant-SKATO [15], STAAR [14], etc., are proposed for rare variant association tests in genomics studies. These methods either do not fully leverage variant functional annotations, or need variant collapsing, with a potential of losing power.

To improve the power in detecting risk genes driven by a sparse number of true risk rare variants and accounting for heterogeneous variant effect, we recently developed a Bayesian–MIRAGE approach [12] for the rare variant association test, which does not need collapsing; rather, it focuses on single variants. MIRAGE is designed for inherited rare variants in family trio samples or rare variants from well-matched case–control data, but it is not able to handle de novo mutations yet. MIRAGE accounts for the heterogeneity of the effect size of rare variants by splitting them into different variant groups, according to their functional annotations, i.e., how likely they are to disrupt the coding protein functions. Within each variant group, rare variants are assumed to share similar effect size; but across different groups, the effect size is assumed to be varying. MIRAGE uses the mixture model to capture the uncertainty that a variant is a risk variant by linking the probability of a variant being a risk variant to its functional annotations. To better estimate the effect size in a variant group, MIRAGE borrows risk information of a gene in the genome. The effect sizes of rare variants—e.g., the proportion of risk variants in every variant group and the probability of a gene that is a risk gene—are reported as the MIRAGE output; based on these information, we can calculate the Bayes factor (BF) for every gene and nominate candidate risk genes by Bayesian FDR.

## 3. Results

### 3.1. MIRAGE Identifies Novel Putative Risk Genes for Schizophrenia

It is known that the pathogenic effects of rare variants are heterogeneous, e.g., the effect size of PTV (protein truncating variants) differs dramatically from missense variants; therefore, ref. [9] splits rare variants into different variant classes, such as PTV and missense variants. Within each variant class, the burden test—which collapses a set of rare variants into a single score—was used to calculate the *p* values. The burden test implicitly assumes that all the rare variants that are to be collapsed are risk variants, which is too strict because most of rare variants could be non-functional [9,10,11]. In this paper, we applied MIRAGE which is powerful in detecting sparse risk variants in the SCHEMA case–control sample using same definitions of variant classes, i.e., three variant groups, PTV, MPC > 3, and MPC 2–3 with an MPC pathogenicity score [16], with the aim of better accounting for the heterogeneity of the variant effect. Furthermore, only ultra-rare variants with a minor allele count no more than 5 are retained for the analysis, as in [9].

Instead of searching through all the genes in the genome-wide database, we restricted the analysis to over 3000 constraint genes with pLI > 0.9 in [17], similarly as in [9], because most genetic signals are believed to be concentrated on constraint genes [18]. Since PTV and missense variants with MPC > 3 show similar burden in SCHEMA analysis, we combined these two variant classes into one group (class I variants), and missense variants (class II variants) with MPC 2–3 were in another group. MIRAGE was run on these two variant classes over constraint genes. The relative risk, $\bar{\gamma }$, of every variant group was determined, as hyperparameters were set at 3, 1 for variant class I and II, respectively, and were used as input to run MIRAGE. The larger the $\bar{\gamma }$, the more likely it is for the variant to a risk variant; MIRAGE is demonstrated to be robust to the choices of hyperparameters as long as they are reasonably close to the underlying true values [12]; these are typically unknown, but could be estimated by empirical studies. More implementation details of MIRAGE are available in [12]. As for class I variants, other choices of hyperparameters, e.g., 4, 5, were also considered, but there were no significant changes in the parameter estimates (see in Supplementary Figure S1); in particular, the proportion of risk genes, δ, and the proportion of risk variants in variant class II, $\eta_{2}$, supporting the robustness of MIRAGE. The hyperparameter of 1 for class II is used because it is not expected to have significant amount of risk variants due to the low MPC score.

**Figure 1:**
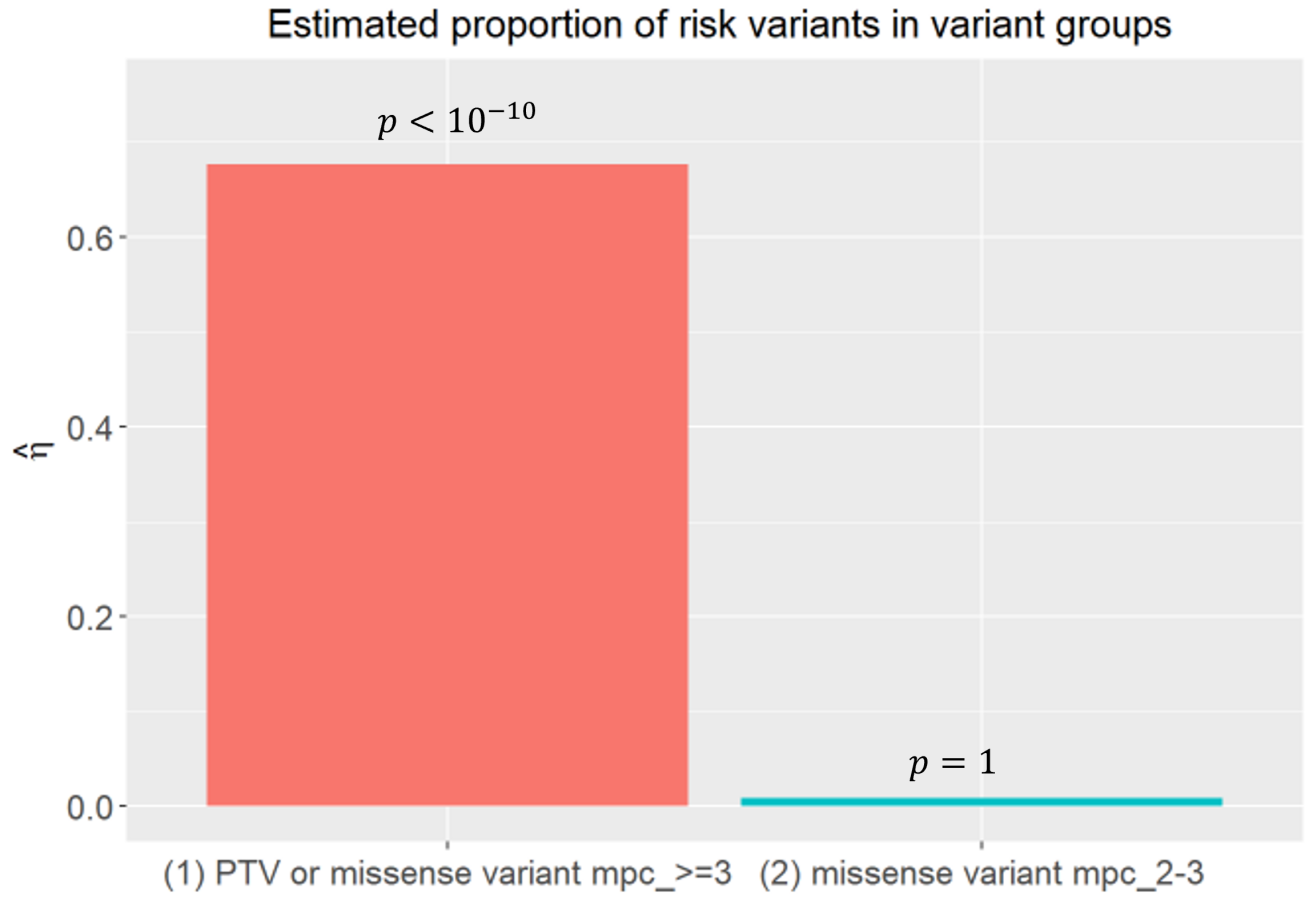
Parameter estimates by MIRAGE, ${\hat{\eta }}^{\prime }s$, the estimated proportion of risk variants in variant groups.

It was estimated by MIRAGE that 45.1% constraint genes, i.e., nearly 1500 genes in total are involved in conferring risk to schizophrenia. Interestingly, this number is consistent with the estimated number of SCZ risk genes in other independent studies [19] and autism risk genes in [20]. As expected, class I variants has as high as 67.66% (with $p<10^{-10}$) variants estimated to be risk variants (Figure 1), while the proportion of risk variants in class II drops substantially to 8.1%, without statistical significance (Figure 1); this suggests that there is no significant amount of risk variants in the class II variant group, which is in agreement with the low burden of variants in this group [9]. Therefore, the Bayes factor of a gene is largely contributed by the class I variant group, which is consistent with the burden results of these two variant groups in the SCHEMA paper [9], where the burden of a gene is mostly from the class I variant group. With the estimated proportion of risk genes, the δ, and the proportion of risk variants in every variant class, $\eta_{1},\eta_{2}$, the Bayes factors of every gene could be calculated by MIRAGE. Bayesian FDR, such as FDR<5%, was used to nominate the candidate risk genes.

### 3.2. MIRAGE Risk Genes Largely Overlapping with Risk Genes in SCHEMA Results

**Figure 2:**
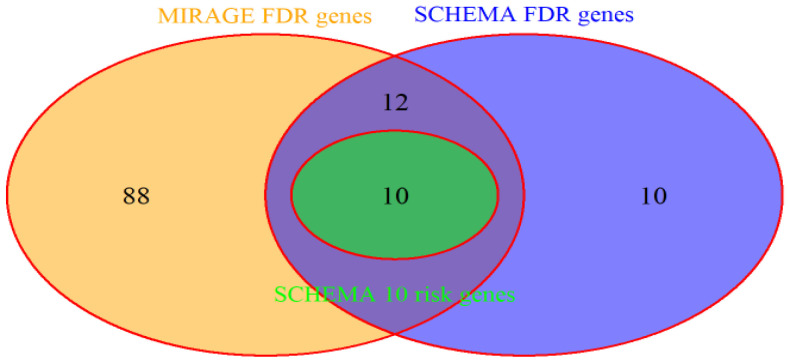
MIRAGE FDR genes, overlapping with 10 SCHEMA risk genes and 32 SCHEMA FDR genes.

We use Bayesian FDR to determine the top risk genes with a large Bayes factor. In [9], 32 genes were selected at FDR<5%, including 10 genes with exome-wide significance. These risk genes are mostly implicated from the case–control sample because de novo samples only take a very small proportion. To make a fair comparison between our results and the SCHEMA findings, we used the top 10 (call SCHEMA 10 risk genes) and 32 (call SCHEMA FDR genes) genes from the case–control sample (throughout and stated otherwise) only without de novo data. Note that these top genes overwhelmingly dominate the list of the top genes from the combined sample in the SCHEMA results due to the small proportion of de novo samples. With the same FDR level, i.e., FDR<5%, MIRAGE implicates 110 genes (we call these the MIRAGE FDR genes), including all 10 SCHEMA risk genes, and interestingly also including 10 risk genes from the combined sample in SCHEMA. Figure 2 is the Venn diagram demonstration of how 110 MIRAGE FDR genes, SCHEMA 10 risk genes, and 32 SCHEMA FDR genes overlap among each other. By the Fisher exact test, significant enrichment was observed between MIRAGE FDR genes and 10 SCHEMA risk genes with *p* value of $3\times 10^{-10}$ and between MIRAGE FDR genes and 32 SCHEMA FDR genes with *p* value less than $2\times 10^{-16}$, i.e., 22 of 32 (22/32 = 68.75%) risk genes overlap with the MIRAGE FDR genes. Moreover, 88 of 110 (80%) MIRAGE FDR genes (we call MIRAGE new genes) are new putative risk genes not identified by SCHEMA burden test. Among 32 SCHEMA FDR genes, 10 genes are not in the MIRAGE FDR genes list, because 8 genes of these 10 missing genes are not in constraint genes [17] and the BF of another 2 genes do not reach desired FDR level. Therefore, MIRAGE FDR genes capture 22 (68.75%) SCHEMA FDR genes, and implicate 88 MIRAGE new genes, at FDR<5%. In the next section, we will investigate the enrichment of new candidate genes and how they are functionally related to schizophrenia.

### 3.3. Gene Set Enrichment of MIRAGE FDR Genes

#### 3.3.1. MIRAGE FDR Genes Significantly Enriched with Autism and Other Disorder Genes

**Figure 3:**
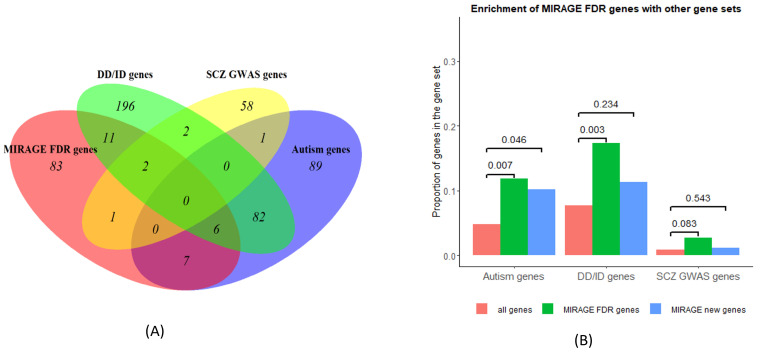
(**A**) MIRAGE FDR genes overlapping with autism, DD/ID, and SCZ GWAS genes. (**B**) Enrichment of MIRAGE FDR/new genes, comparing to all constraint genes, with Fisher exact test *p* values shown over the bars.

Evidence suggests that genetic signals are shared across schizophrenia, autism, and developmental disorders [9,21,22], so it is interesting to investigate how MIRAGE FDR genes are enriched with autism and other disorder genes. It turns out that, among 110 MIRAGE FDR genes for SCZ, 13 appear in 102 (13/102 = 12.75%) autism genes [23]; 19 are overlapping with 299 (6.35%) DD/ID genes (developmental delay/intellectual disability); 3 genes are in the list of 64 (4.69%) SCZ GWAS genes (Figure 3A, Table 1). These extend the shared gene list in [9]. From the enrichment in Figure 3B, the MIRAGE FDR genes are significantly enriched in autism genes and DD/ID genes, but not in SCZ GWAS genes, as determined by Fisher exact test. After excluding 22 SCHEMA FDR genes, the remaining 88 MIRAGE new genes are only enriched with autism genes, not in DD/ID genes and SCZ GWAS genes; thus, the enrichment is largely driven by the SCHEMA FDR genes and the MIRAGE new genes are significantly enriched with autism genes only. Therefore, this analysis provides evidence of shared risk genes between autism and schizophrenia; again, it was hard to find SCZ-overlapping genes between the MIRAGE FDR genes from URV and the SCZ GWAS genes from common variants [9].

We list the shared genes with autism, DD/ID genes, and SCZ GWAS genes in Table 1, where genes outside the box are from MIRAGE new genes. Evidence in the literature suggests that the new shared genes are potential SCZ risk genes. For instance, gene DSCAM is involved in contributing to nervous system in schizophrenia with 22q11.2 deletion syndrome [24]; SHANK3 is a known autism gene and mutations in this gene also appear in the schizophrenia phenotype [25]; the ZMYND11 gene is implicated in complex neuropsychiatric features [26]; ANK2 is essential for neuronal morphogenesis and long term memory in drosophila studies [27]. Multiple lines of evidence implicate deletions in gene NRXN1 with BF of 46.31, implying that it is related to conferring a risk of schizophrenia [28,29,30,31]. The coding protein of gene PCDH19 plays an important role in brain development [32,33]. Gene CLTC is found to be associated with childhood-onset schizophrenia in family trio/duo sample [34]. Gene CACNA1C is a susceptibility gene for schizophrenia according to meta-analysis and GWAS [35,36]. In this study, we provide statistical evidence that these genes could be schizophrenia putative risk genes.

#### 3.3.2. GO Enrichment Analysis

We performed GO enrichment for the MIRAGE FDR genes and the MIRAGE new genes powered by PANTHER (https://geneontology.org/, accessed on 17 October 2023), with the genome-wide gene set as the reference. After Bonferroni correction, we found significant enrichment in GO biological process, GO cellular component, and many others. To demonstrate the enrichment that is not due to 22 SCHEMA FDR genes alone, we conducted GO enrichment for 88 MIRAGE new genes (Table 2) as well and more details are available in the Supplementary Excel. Most top enrichment instances are from 110 MIRAGE FDR genes, with 22 SCHEMA FDR genes included and a small number of enrichment instances are from 88 MIRAGE new genes by comparing the fold changes between them.

In the complete GO biological process, membrane depolarization during cardiac muscle cell action potential (GO:0086012), mechanosensory behavior (GO:0007638), membrane depolarization during action potential (GO:0086010), cell–cell signaling involved in cardiac conduction (GO:0086019), and regulation of heart rate by cardiac conduction (GO:0086091) have more than 10-fold changes for the 110 MIRAGE FDR genes with statistical significance. As for 88 MIRAGE new genes, mechanosensory behavior (GO:0007638) has increased fold change by 10, suggesting these new genes are more enriched in mechanosensory behavior which are linked to schizophrenia [37,38,39]. Other enriched items with more than 10-fold changes disappear for 88 MIRAGE new genes, indicating these enrichment instances are driven by 22 SCHEMA FDR genes. The enrichment of 88 MIRAGE new genes includes the regulation of cell size (GO:0008361) with a fold change of over 9; neuron projection morphogenesis (GO:0048812), plasma-membrane-bounded cell projection morphogenesis (GO:0120039), cell projection morphogenesis (GO:0048858) and cell part morphogenesis (GO:0032990) all have fold changes over 5. Interestingly, there is a rich line of evidence showing the involvement of these biological processes in schizophrenia. For example, studies found reduced pyramidal cell somal volume in specific brain areas and reduced hippocampal volume for subjects with schizophrenia [40,41]. Abnormality of glutamatergic projection neurons and multiple classes of GABA-ergic inhibitory neurons are found in schizophrenia [42,43,44,45]. The membrane hypothesis of schizophrenia dates back by decades [46], and recent evidence finds red blood cell membrane lipids are associated with schizophrenia [47].

In the complete GO molecular function, voltage-gated calcium channel activity involved in cardiac muscle cell action potential (GO:0086007) has dramatic enrichment, with more than 100 fold changes for 110 MIRAGE FDR genes, but this disappears for 88 MIRAGE new genes. Therefore, 22 SCHEMA FDR genes contribute solely to this enrichment. There is a rich body of the literature about the association between voltage-gated calcium channels and psychiatric disorders [48,49,50]. Instead, RNA binding is slightly more enriched in the 88 MIRAGE new genes than in the 110 MIRAGE FDR genes.

In the complete GO cellular function, the top enriched components include pre-synaptic active zones (GO:0048786), GABA-ergic synapse GO:0098982), postsynaptic density GO:0014069), asymmetric synapse (GO:0032279), neuron to neuron synapse (GO:0098984), postsynaptic specialization (GO:0099572), postsynaptic (GO:0098794), and glutamatergic synapse (GO:0098978) for 110 MIRAGE FDR genes. This is not the case for the 88 MIRAGE new genes. However, neuronal cell body (GO:0043025), cell body (GO:0044297), and pre-synapse (GO:0098793) are enriched in 88 MIRAGE new genes only. Links between cell body and schizophrenia can be found in the literature [51]. Comparing to 110 MIRAGE FDR genes, import into cell (GO:0098657) and nervous system development (R-HSA- 9675108) are slightly more enriched in 88 MIRAGE new genes; this is supported by evidence in multiple studies [52,53]. Therefore, putting all our findings together, MIRAGE new genes suggest distinct biological functions from SCHEMA FDR genes, providing new insights into the mechanisms of schizophrenia.

## 4. Discussion and Conclusions

Rare variant association analysis is challenging since it is under-powered. In this study, we applied a Bayesian rare variant association test method, MIRAGE to SCHEMA case–control sample, and identified more novel candidate risk genes associated with schizophrenia, with all 10 risk genes reported in SCHEMA being captured [9], further enhancing the risk roles of these genes. The identification of 88 new SCZ risk genes is attributable to the power of MIRAGE in better accounting for the heterogeneous variant effect and capturing sparse signal in rare variants. Consistent with the findings in [9], PTV and missense variants with MPC≥3 carry more burden with statistical significance than missense variants with MPC < 3. More interestingly, a total of about 1500 genes are estimated to be involved in the pathogenesis of schizophrenia, agreeing with the numbers reported in other studies [19]. MIRAGE FDR genes are significantly enriched in autism and DD/ID risk genes, but not in SCZ GWAS genes, consistent with findings in [9], which again indicates some degree of convergence in the genes implicated by common loci and ultra-rare variants. However, MIRAGE new genes significantly overlaps with autism genes only with less statistical significance, indicating these new candidate risk genes carry fewer overlaps than SCHEMA FDR genes in autism genes, DD/ID, and SCZ GWAS genes, which indicates how risk genes are shared across different neurodevelopmental disorders.

From the GO enrichment analysis, few enrichment instances are shared between MIRAGE FDR genes (including 22 SCHEMA FDR genes) and MIRAGE new genes (without 22 SCHEMA FDR genes), which implies their distinctive biological functions. Among the shared enrichment, some are enhanced from MIRAGE FDR genes to MIRAGE new genes, such as mechanosensory behavior (GO:0007638), and others are weakened, such as somatodendritic compartment (GO:0036477). However, many enriched items in MIRAGE FDR genes becomes weaker or even disappear in MIRAGE new genes, and some enrichment in MIRAGE new genes are not found in MIRAGE FDR genes, which is indicative of different molecular functional features between SCHEMA FDR genes and MIRAGE new candidate genes. Therefore, MIRAGE new genes may provide new insights on the etiology of schizophrenia. However, it needs to be noted that MIRAGE new genes are based on statistical association evidence, so animal models are required to justify their functional roles on the development of schizophrenia.

In the future, it would be interesting to integrate de novo samples to prioritize SCZ risk genes, like TADA [20], using DeNovoWEST [54], especially when a large number of de novo samples of SCZ become available. It is also of interest to study the temporal expression of MIRAGE new genes in the brain using bulk RNA-seq data from BrainSpan [55], and single cells from the mouse nervous system [56], or integrate genetics signal and gene expression to gain more power in the identification of novel schizophrenia risk genes.

## Figures and Tables

**Table 1:** MIRAGE FDR genes overlapping with autism genes, DD/ID genes, and SCZ GWAS genes. Genes in boxes are in 32 SCHEMA FDR genes and outside are new candidate genes of schizophrenia.

Autism Genes	DD/ID Genes	SCZ GWAS Genes
ITSN1; DSCAM; ZMYM2; SHANK3; FAM120A; ZMYND11; KDM6B; ANK2; ASH1L; CAMTA2; NRXN1; ADCY5; PSMD11	ZMYM2; SHANK3; STAG1; GRIN2A; ZMYND11; KDM6B; PCDH19; HIVEP2; TRIO; ASH1L; SETD1A; SRRM2; CACNA1G; PPP2R1A; BMPR2; CLTC; ADCY5; CACNA1C; SATB1	SP4; GRIN2A; CACNA1C

**Table 2:** Fold changes with *p* values in parenthesis of top enrichment in GO enrichment analysis. “-” represents NA.

		MIRAGE FDR (110) Genes	MIRAGE New (88) Genes
Complete GO biological process	Membrane depolarization during cardiac muscle cell action potential (GO:0086012)	53.13 (0.0011)	-
	Mechanosensory behavior (GO:0007638)	51.61 (0.0233)	64.65 (0.0094)
	Membrane depolarization during action potential (GO:0086010)	47.53 (0.0017)	-
	Cell–cell signaling involved in cardiac conduction (GO:0086019)	33.45 (0.0077)	-
	Regulation of heart rate by cardiac conduction (GO:0086091)	22.03 (0.0491)	-
	Regulation of cell size (GO:0008361)	-	9.28 (0.0292)
	Neuron projection morphogenesis (GO:0048812)	-	5.38 (0.0253)
	Plasma-membrane-bounded cell projection morphogenesis (GO:0120039)	-	5.32 (0.0279)
	Cell projection morphogenesis (GO:0048858)	-	5.27 (0.0308)
	Cell part morphogenesis (GO:0032990)	-	5.08 (0.045)
Complete GO molecular function	Voltage-gated calcium channel activity involved in cardiac muscle cell action potential (GO:0086007)	>100 (0.0272)	-
	RNA binding (GO:0003723)	2.59 (0.0436)	2.83 (0.0365)
Complete GO cellular component	Pre-synaptic active zone (GO:0048786)	13.06 (0.0152)	-
	GABA-ergic synapse (GO:0098982)	12.18 (0.0221)	-
	Postsynaptic density (GO:0014069)	6.28 (0.0010)	-
	Asymmetric synapse (GO:0032279)	6.04 (0.0015)	-
	Neuron to neuron synapse (GO:0098984)	6.02 (0.0015)	-
	Postsynaptic specialization (GO:0099572)	5.87 (0.0020)	-
	Postsynapse (GO:0098794)	5.16 (<0.0001)	
	Glutamatergic synapse (GO:0098978)	5.03 (0.0095)	-
	Neuronal cell body (GO:0043025)	-	4.98 (0.0234)
	Synaptic membrane (GO:0097060)	4.97 (0.0264)	-
	Dendrite (GO:0030425)	4.87 (0.0001)	-
	Dendritic tree (GO:0097447)	4.86 (0.0002)	-
	Cell body (GO:0044297)	-	4.79 (0.0136)
	Somatodendritic compartment (GO:0036477)	4.38 (<0.0001)	4.25 (0.0017)
	Pre-synapse (GO:0098793)	4.11 (0.0311)	-
PANTHER GO-Slim Biological Process	Import into cell (GO:0098657)	6.18 (0.0362)	6.88 (0.0474)
Reactome pathways	Neuronal System (R-HSA-112316)	4.94 (0.0409)	-
	Nervous system development (R-HSA-9675108)	4.4 (0.0103)	4.72 (0.023)
	Axon guidance (R-HSA-422475)	4.27 (0.0314)	-

## Data Availability

The datasets analyzed during the current study are available in the SCHEMA website [https://schema.broadinstitute.org/] (accessed on 02 May 2023).

## Supplementary Materials

The following supporting information can be downloaded at: https://www.mdpi.com/article/10.3390/jpm14080822/s1.

## Abbreviations

The following abbreviations are used in this manuscript:

SCHEMA	schizophrenia exome meta-analysis
SCZ	schizophrenia
GWAS	genome-wide association study
SNV	single nucleotide variants
CNV	copy number variation
FDR	false discovery rate
URV	ultra-rare variants
MIRAGE	mixture model based rare variant analysis on genes
MAC	minor allele counts
GO	gene ontology
