# Supplementary figures and images for: Bayesian Rare Variant Analysis Identifies Novel Schizophrenia Putative Risk Genes
Source: J Pers Med. 2024 Aug 2;14(8):822. doi: 10.3390/jpm14080822 (PMC11355493; doi:10.3390/jpm14080822)

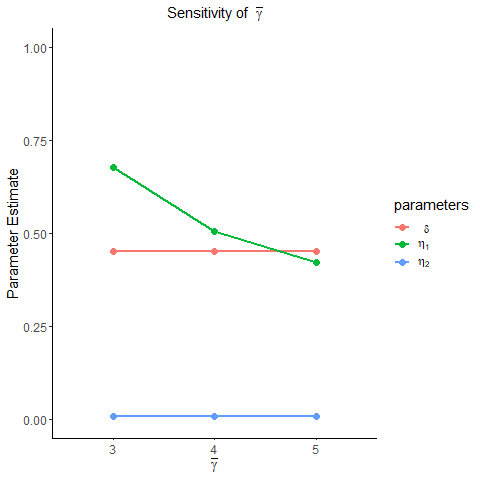

Supplement: Supplementary file 1 [file jpm-14-00822-s001.zip › jpm-3088733-figures.png]
